# Durable ZrB_2_–ZrC Composite Materials as Advanced Electrodes for High-Performance Supercapacitors

**DOI:** 10.1021/acsomega.5c01560

**Published:** 2025-04-25

**Authors:** Aybike Paksoy, Ahmet Güngör, İpek Deniz Yıldırım, Seyedehnegar Arabi, Emre Erdem, Özge Balcı-Çağıran

**Affiliations:** † Koç University Boron and Advanced Materials Application and Research Center (KUBAM), Rumelifeneri Yolu, Sarıyer, Istanbul 34450, Türkiye; ‡ Faculty of Engineering and Natural Sciences, Sabancı University, Orhanlı, Tuzla, İstanbul 34956, Türkiye; § Industrial Systems Engineering Department, University of Regina, Regina S4S 0A2, Canada; ∥ Department of Materials Science and Engineering, İzmir Institute of Technology, Urla, İzmir 35430, Türkiye

## Abstract

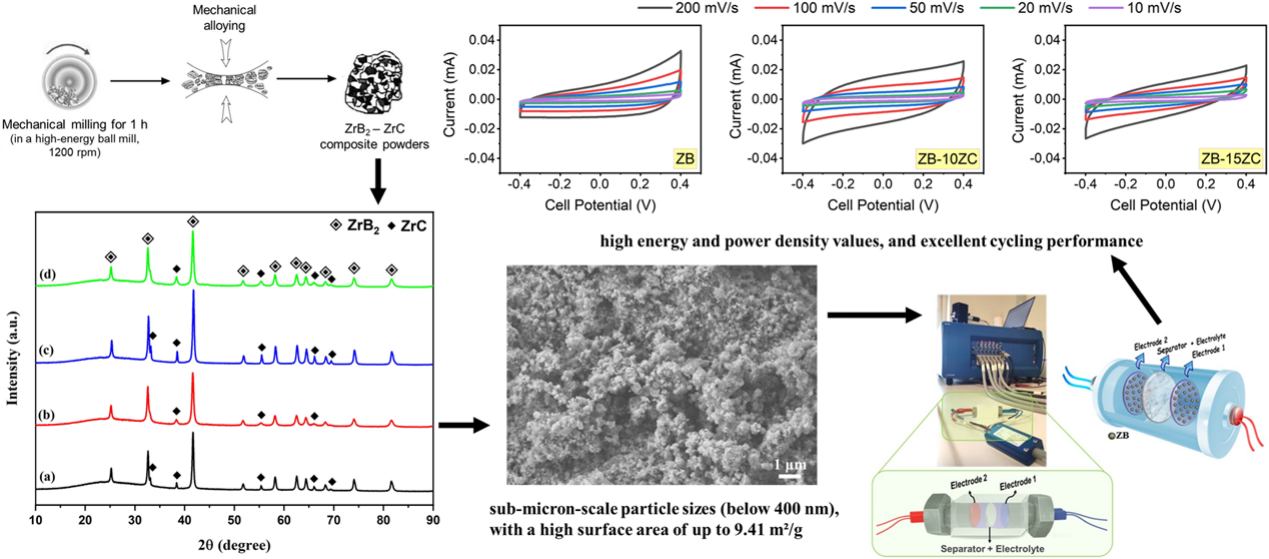

Boride and carbide-based materials attract increasing attention as promising options for energy storage applications. This research focuses on synthesizing pure boride and carbide compounds of zirconium (ZrB_2_ and ZrC) and their composite powders using mechanical activation-assisted route and subsequent heating processes. The chemical and microstructural characterization results indicate that the synthesized composite powders are of high purity, possess submicron-scale particle sizes (below 400 nm), and exhibit a high surface area of up to 9.41 m^2^/g. Supercapacitor devices, using the resulting powders as symmetrical electrodes, exhibit high energy density values ranging from 5.8 to 8.8 Wh/kg. The ZrB_2_–15 wt % ZrC composite sample achieves the highest power density at 155 W/kg, compared to 118 W/kg for the pure ZrB_2_ sample. Cycling tests demonstrate exceptional capacitance retention (99.4–99.9%) and cyclic stability, even after 5000 cycles, highlighting the high durability of the composite samples. These findings show that ZrB_2_–ZrC composites exhibit high energy and power density values and excellent cycling performance, making them strong candidates for use in high–performance supercapacitor devices.

## 1 Introduction

Energy production has long relied on fossil fuels, which are still used, but with their limitations and a growing population, efficient energy production and storage are becoming increasingly crucial.^1,2^ Various studies have been conducted, and devices have been developed to produce and store electrical energy. Batteries and fuel cells, the most widely used electricity-generating devices, benefit from the ion exchanges of two poles (anode and cathode) in an electrolytic solution; they retain chemical energy in their structures and convert it into electrical energy. Conventional capacitors, another important energy source, although they have a similar structure to batteries, do not store chemical energy but store potential energy electrostatically on the electrode surfaces.^1,2^ In both cases, during charge–discharge, ions move between the electrodes called anode–cathode or positive–negative.^3^ However, it can be said that a significant disadvantage of batteries, fuel cells and traditional capacitors is that the amount of charge–discharge (known as the number of cycles) is limited. This situation causes situations such as constantly extending the charging time and shortening the discharge time since the energy source starts to be used.^2^ Due to these problems in current technologies, scientists continue to work on a technology called supercapacitor (SC), also known as ultracapacitor or electrochemical double-layer capacitors (EDCLs).^1^ The idea of supercapacitors (SCs) is not new. In their study published in 1853, Helmholtz and his colleagues showed that it is not necessary to store energy only on the surface of conductive electrodes but that it is also possible with the formation of a double layer between the electrode and the electrolyte.^4^ The main difference of SCs from existing technologies is the formation of an electrical deposition at the interface between the electrolyte solution and the electrode material.^5,6^ SCs are called symmetrical or asymmetrical depending on whether the electrodes on both sides are made of the same material.^5^

Supercapacitors, often regarded as revolutionary in the energy sector, offer the advantage of high power density but face a notable drawback in their low energy density. Since this problem is thought to be solved by changing the electrode material, researchers continue their studies on different electrode materials.^7^ When we look at electrode materials, we see zero-dimensional (0D), one-dimensional (1D), two-dimensional (2D) and three-dimensional (3D) materials, as is the basis of nanotechnology.^8^ Among zero-dimensional powder materials, a lot of research has been done so far, especially on carbon nanomaterials (such as activated carbon and mesoporous carbon) and oxide forms of transition metals (such as NiO, MnO).^8−11^ Unlike existing studies, metal borides have attracted the most attention recently due to their electronic conductivity, layered structure and size that can into the interstitial sites of transition metals.^7,12^ There are studies in the literature on metal borides such as CoB, NiCoB, MoB, and HfB_2_.^7^ In this study, we search for the use of electrode material for some rare investigated materials such as metal borides (like zirconium diboride), boride-carbide composites, and metal carbides.

Carefully examining the parameters such as conductivity value, surface morphology, specific surface area, and porosity of the materials to be used in the electrodes of SCs and selecting the appropriate material directly affects the performance of SCs. For this reason, synthesizing new materials that are candidates for use as electrodes is of vital importance.^13^ Many researchers initially tried to use oxide forms of transition metals as electrode materials of SCs since they are both readily available in nature and can be easily prepared in the laboratory environment. However, the low electronic conductivity of transition metal oxides has led researchers to investigate sulfides, nitrides, carbides, selenides, phosphides and borides.^7,12,14−20^ Although oxide-based supercapacitors (SCs) are known for their low electrical conductivity, significant progress has been made to address this limitation. Strategies such as doping with conductive elements, nanostructuring to reduce charge transport distances, and hybridization with highly conductive materials like graphene or carbon nanotubes have shown promising results in enhancing their conductivity and overall performance.^21^ These advancements have expanded the applicability of oxide-based SCs, yet challenges remain, prompting researchers to explore alternative materials such as sulfides, nitrides, carbides, and borides.^22^ The unique electronic structure of boron allows the formation of both covalent and metallic-like bonds.^23,24^ Transition metal borides (TMBs) have attracted attention for SCs due to their metal–metal, metal-B and B–B bonding. TMBs have critical physical properties such as thermal and chemical stability, high electrical conductivity, high specific capacitance value and electrochemical stability.^14^ Among TMBs, ZrB_2_ is an ultrahigh temperature ceramic (UHTC) with a very high melting temperature (3245 °C), high thermal conductivity (57.9 Wm^1–^K^–1^), high electrical conductivity, low thermal expansion rate (5.9 × 10^–6^ °C^1–^), high thermal shock resistance, high chemical stability, high hardness (23 GPa) and high corrosion resistance. Due to these important properties, they are used in refractories, electronic devices and hypersonic spacecraft.^25−27^ Considering all the reasons mentioned, using ZrB_2_ as a supercapacitor electrode may be beneficial in providing the features expected from a supercapacitor. Paksoy et al., in their study published in 2023, produced symmetrical and asymmetrical ZrB_2_ supercapacitor electrodes by mechanical activation-assisted direct synthesis method and performed their electrochemical analysis. As a result of their work, after 50 cycles, the symmetric supercapacitor exhibited 79% capacitance retention, and the asymmetric supercapacitor exhibited 69% capacitance retention.^7^ Carbides, another important material group, are generally examined in four groups: those consisting of isolated carbon atoms, those consisting of isolated carbon atom pairs, those consisting of carbon atom chains, and finally, those consisting of carbon atom networks.^28^ Although there are structures consisting only of carbon atoms (such as graphite and graphene), transition metal carbides (MCs) have also been investigated many times, especially due to their very high mechanical strength.^29−32^ Balcı et al. investigated boron carbide as SC electrode materials, and obtained 58 Wh kg^–1^ energy density, 2000 mAh g^–1^ specific capacity and 96.9 F g^–1^ specific capacitance values at 0.1 A g^–1^ current density. At the end of the study, they reported that B_4_C composites could be used as SC electrodes and stated that reducing the particle size would provide better electronic properties.^31^ Thirumal et al. studied using antimony/titanium carbide (Sb/Ti_3_C_2_T_*x*_) MXenes as SC electrode materials. They reported that the electrodes they prepared showed a specific capacitance of 184.72 F g^–1^ and an energy density of 53.19 Wh kg^–1^ at a current density of 1 A g^–1^. They also stated a high capacitance retention of 91.23% after 10,000 charge–discharge cycles.^33^ Very few studies in the literature on supercapacitor production use transition metal borides and carbides together. One of the most recent publications in our previous study is the HfB_2_–SiC symmetric supercapacitors of Paksoy et al.^34^ We reported that pure HfB_2_ electrodes exhibited the highest power density (95.23 W kg^–1^), whereas the electrode containing 15% SiC exhibited a power density of 75.30 W kg^–1^.^34^ In another recent study, Hao et al. created a composite structure with SiC to increase the relatively low toughness of a high entropy diboride with high hardness, high-temperature strength and superior oxidation resistance. For this purpose, they produced (Ti_0.2_Zr_0.2_Hf_0.2_Nb_0.2_Ta_0.2_)B_2_–SiC composite and proposed it to be used as an SC electrode.^35^

In this study, we detailed the synthesis methods for pure zirconium boride (ZrB_2_) and zirconium carbide (ZrC) compounds and their composite structures. We documented the electrochemical properties of these materials used in symmetric supercapacitor devices, providing insights into their performance as electrodes. In this study, a two-electrode system was employed for electrochemical analysis to evaluate the performance of the fabricated symmetric supercapacitors under practical working conditions. This configuration provides a realistic assessment of the device-level performance, including the combined contributions of both electrodes and the electrolyte. While a three-electrode system is often used to investigate the intrinsic properties of individual electrodes, our focus was on the overall performance of the symmetric supercapacitor devices. This work aims to contribute concrete experimental data on the behavior of boride–carbide composites in supercapacitors to the existing literature.

## 2 Experimental Procedure

### 2.1 Synthesis and Characterization of Pure ZrB_2_ and ZrC and Their Composite Powders

In this study, we first synthesized ZrB_2_ and ZrC powders separately using mechanical activation-assisted direct synthesis routes and then combined them to create composite structures. To produce pure ZrB_2_ powders, the powder mixtures of zirconium tetrachloride (ZrCl_4_, Alfa Aesar, 99%), nanoboron (amorphous nano-B, PavTec, 99%), and magnesium (Mg, Alfa Aesar, 99.8%) were used as precursors with a respective mole ratio of 1:2:1. The ZrB_2_ powders used in this study were synthesized following the same parameters as described in our previous work.^7^ The detailed experimental procedures are provided in that publication. To produce pure ZrC powders, the powder mixtures of zirconium oxide (ZrO_2_, Alfa Aesar, 99%) and graphite (C, Alfa Aesar, 99.8%) were used as precursors with a respective mole ratio of 1:3. The powder mixtures were first mechanically milled for 3 h and then reacted at 1500 °C (for a duration period of 6 h) in a tube furnace under Ar gas flow. Table 1 shows the synthesis conditions of ZrB_2_ and ZrC samples, named ZB and ZC, respectively. All mechanical milling processes in this study were carried out using a Spex 8000D high-energy ball mill with a rate of 1200 rpm. Stainless steel milling vials and balls were used for this process, and the ball-to-powder ratio was determined as 10:1. To prevent oxidation reaction during the process, the pellet-shaped samples were produced using the cold-pressing technique in an MBraun glovebox, executed under an Ar atmosphere. Subsequently, the prepared pellets were used for chemical reaction purposes.

**Table 1:** Synthesis Conditions of ZrB_2_ and ZrC Powders

	synthesis conditions			
powder name	starting materials (powder)	mechanical activation	mole ratio (mole)	reaction temperature (°C)
ZrB_2_	ZrCl_4_–B −Mg	2 min	1:2:1	850
ZrC	ZrO_2_–C (graphite)	3 h	1:3	1500

**Figure 1:**
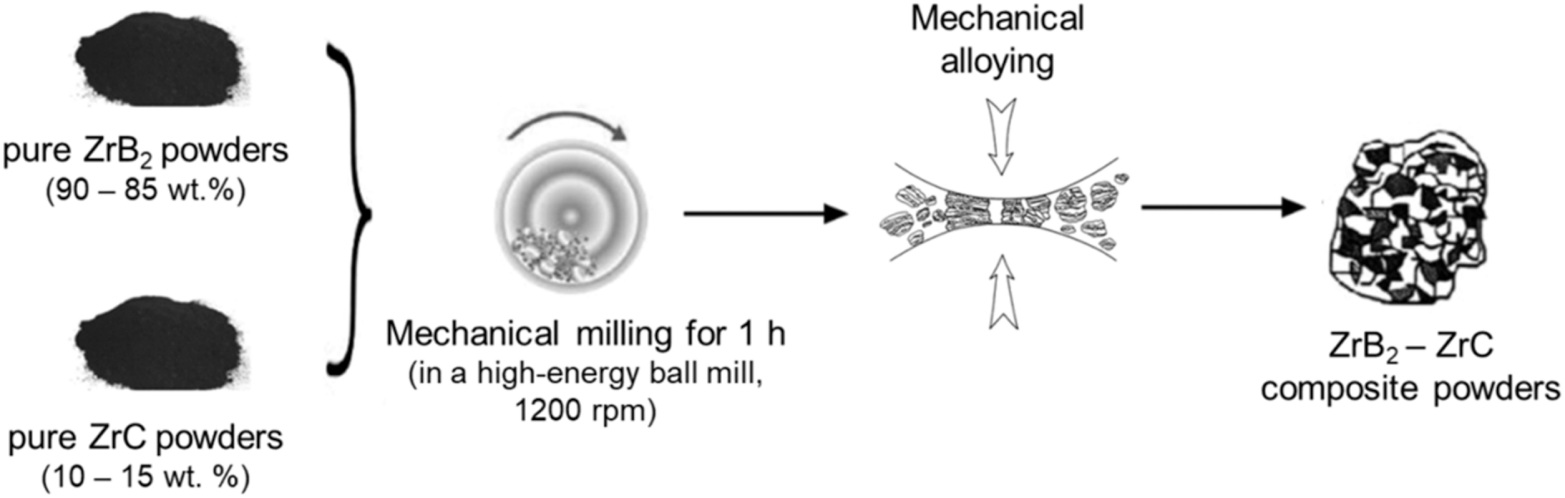
Schematic representation of the mechanical alloying process used in this study.

A mechanical alloying process was used to produce composite structures, and a schematic representation of this process is provided in Figure 1. The synthesized ZrB_2_ powders have been mechanically milled with 10 or 15 wt % of ZrC ratios to generate the desired composite samples. Thus, two distinct composite samples, including 10 wt % ZrC–90 wt % ZrB2 and 15 wt % ZrC–85 wt % ZrB2, prepared in the glovebox under an Ar gas atmosphere, were named ZB-10ZC and ZB-15ZC, respectively.

A Rigaku Miniflex600 Series X-ray diffractometer (XRD) using Cu Kα radiation was used for the phase analysis, with a scan rate of 10°/min and a step size of 0.02°. The Crystallography Open Database (COD) powder diffraction database identified the crystalline phases. The powders’ microstructural and morphological properties were examined using a Bruker Xflash 5010 energy dispersive X-ray spectrometer and a Zeiss Ultra Plus field emission scanning electron microscope (FE-SEM) with a spectral resolution of 123 eV. Particle size distribution graphs were obtained by using a Malvern Zetasizer dynamic light scattering (DLS). Surface area measurements were conducted using the Brunauer–Emmett–Teller (BET) method (Micromeritics Gemini VII) with nitrogen adsorption at −196 °C, a relative pressure range of 0.05–0.3 *P*/*P*_0_, and degassing at 120 °C for 1 h under vacuum. Differential thermal analysis/thermogravimetric (DTA/TG, STA449F3, Netzsch) equipment was used for thermal analyses. DTA/TG analyses were conducted in an alumina crucible heated to 850 °C with a 10 K/min heating rate under an Ar atmosphere.

### 2.2 Electrochemical Performance of Synthesized Samples in Supercapacitors

**Figure 2:**
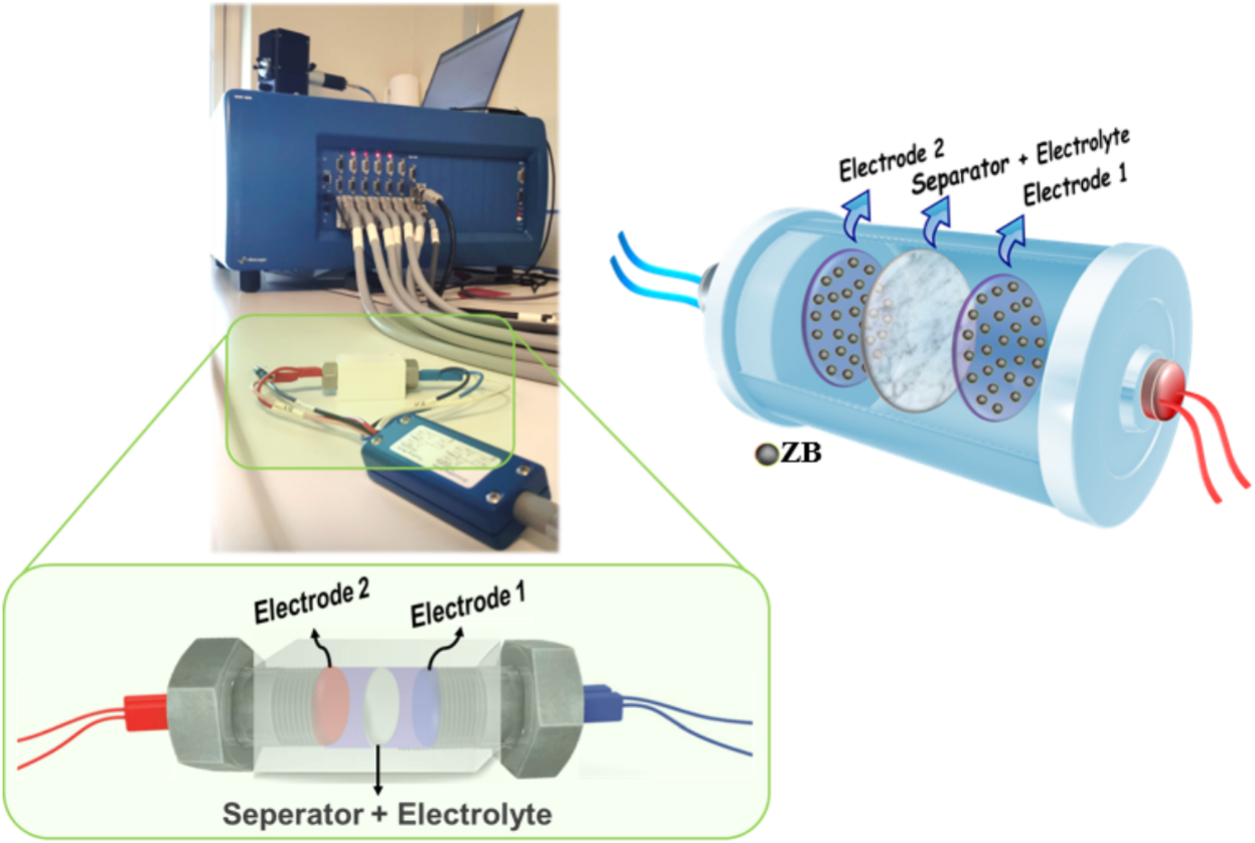
Supercapacitor design with the usual two-electrode configuration.

The potentiostat (BioLogic VMP 300) was applied to test electrochemical performance in a 6 M KOH aqueous solution. Figure 2 demonstrates a graphical representation of the two-electrode system created using the synthesized powders. Figure 2 shows the supercapacitor scheme, which includes two electrodes (Electrode I and Electrode II) that can be made of the same material for a symmetric supercapacitor. The electrode mass loading for the supercapacitor tests was approximately 0.5–1.0 mg per electrode. The reported energy and power densities were normalized to the active material mass of the electrodes, which includes the synthesized powders. This approach highlights the intrinsic performance of the active materials. The separator was glass fiber in whole supercapacitor designs. For evaluating the electrochemical properties, fabricated symmetric supercapacitors were assembled with ZB, ZB-10ZC, and ZB-15ZC composite powder electrodes, respectively. Table 2 shows the cell components of supercapacitors based on ZrB_2_ and ZrB_2_–ZrC composites. Cyclic voltammetry (CV), electrochemical impedance spectroscopy (PEIS), and galvanostatic charge–discharge tests (GCPL) were carried out at room temperature. CV curves were obtained using potentials ranging from −0.4 to 0.4 V and scan rates ranging from 10 to 200 mV/s. GCPL curves were recorded by scanning the potential with current densities of 0.10, 0.50, and 2.40 A/g.

**Table 2:** Cell Components of Supercapacitors Based on ZrB_2_ and ZrB_2_–ZrC Composites

sample name	electrodes 1 and 2	electrolyte	separator	type
ZB	ZB	6 M KOH	glass fiber	symmetric
ZB-10ZC	ZB-10ZC	6 M KOH	glass fiber	symmetric
ZB-15ZC	ZB-15ZC	6 M KOH	glass fiber	symmetric

## 3 Results and Discussion

### 3.1 Microstructural Properties

**Figure 3:**
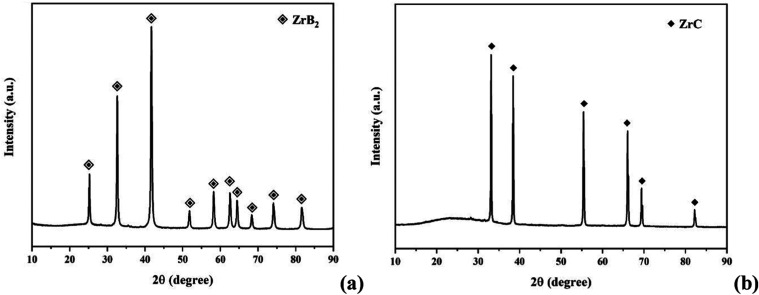
XRD patterns of the synthesized ZB and ZC samples show the pure phases of (a) ZrB_2_ and (b) ZrC.

The final XRD patterns of the synthesized ZrB_2_ and ZrC powders are presented in Figure 3. As seen in Figure 3, pure ZrB_2_ (COD Card Number: 1510857, Hexagonal, *a* = *b* = 3.17 Å, *c* = 3.53 Å) and ZrC (COD Card Number: 9008777, Cubic, *a* = *b* = *c* = 4.68 Å) phases are detected in Figure 3a,b, respectively. The XRD analysis revealed that the material’s crystal structure consists solely of the intended phases. Thermochemical calculations for the stoichiometric reaction between ZrO_2_ (1 mol) and C (3 mol) indicate that ZrC formation begins around 1650 °C. However, in our synthesis experiments, the complete reaction and final formation of the pure ZrC phase were achieved at 1500 °C due to the mechanical activation process applied before the carbothermal reaction. Elevated temperatures lead to the formation of smaller particle sizes, which is crucial for the subsequent use of the powders in preparing composite structures.

**Figure 4:**
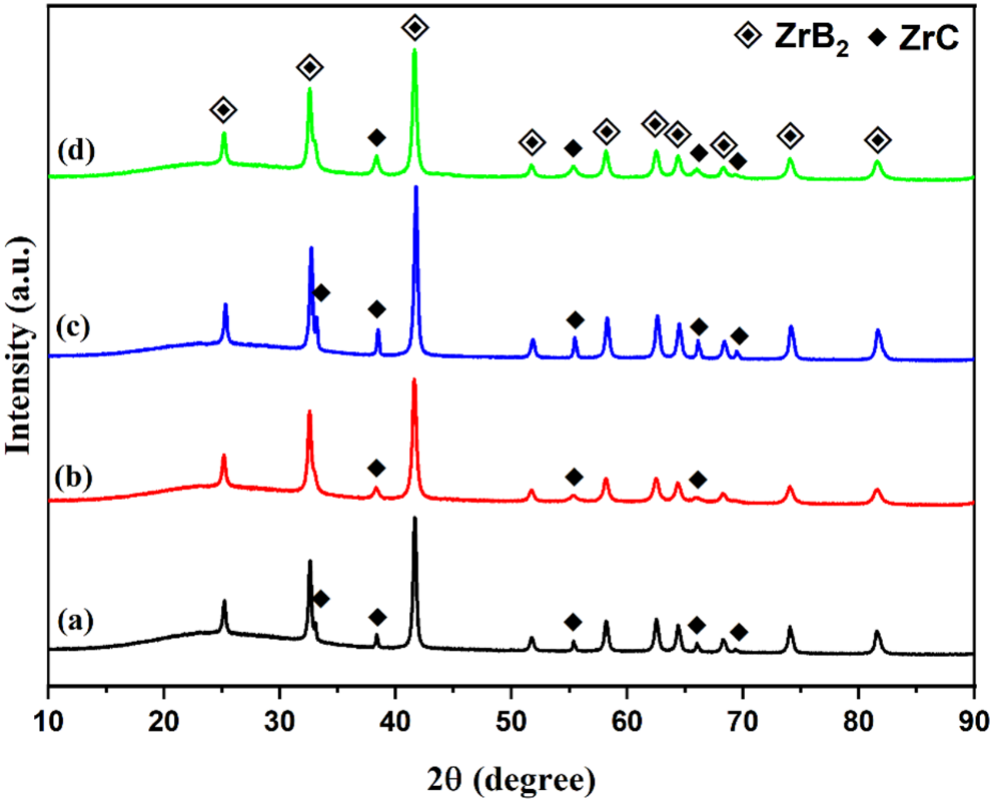
XRD patterns of the synthesized composite powders before and after mechanical alloying: (a) ZB–10ZC before milling; (b) ZB–10ZC after milling; (c) ZB–15ZC before milling; and (d) ZB–15ZC, after milling.

Figure 4 shows the XRD patterns of the synthesized composite powders before and after mechanical alloying. The synthesized ZrB_2_ and ZrC phases were first mixed in an agate mortar, with their XRD patterns presented in Figure 4a,c as “before milling”. As expected, the XRD patterns of the samples display both ZrB_2_ and ZrC phases. After the mechanical alloying process, a significant change in the XRD pattern is observed, particularly at around 2θ = 32°, where the superposition of the ZrB_2_ and ZrC phases indicates the formation of the composite structure. Additionally, the intensity of the other peaks corresponding to the ZrC phase decreases after the mechanical alloying process. Notably, no reactions between the components or the presence of impurity phases were detected in the composite powders due to the ball milling process, within the measurement limits of the XRD technique (≥2 wt %). DTA/TG analyses were performed to further confirm the purity and thermal stability of the synthesized powders, as presented in Figure S1 of the Supporting Information. Due to the high sensitivity of DTA measurements to phase transitions, melting, or oxidation reactions, the presence of impurities such as Mg, C, or unreacted precursors would typically result in distinct thermal events. However, the absence of any exothermic or endothermic peaks in the DTA curve up to 850 °C suggests that no such transitions occur, implying that the powder is free from secondary phases or low-melting-point contaminants. Likewise, the TG curve displays no detectable weight change across the entire temperature range, confirming the absence of volatile species or thermally unstable components. These results collectively confirm the chemical purity, compositional homogeneity, and thermal stability of the final powders with no evidence of residual volatiles, adsorbed species, or unreacted precursors. Figure S2 (in the Supporting Information file) shows the SEM/EDX analysis of the synthesized ZB–10ZC composite powders, indicating the presence of Zr, B, and C in the final powders. This analysis confirms the composite structure and its purity and reveals that two different phases coexist throughout the microstructure. As a result, it is important to highlight the high purity of the synthesized composite powders, as their purity level can significantly influence the electrochemical performance of supercapacitor electrode materials.

**Figure 5:**
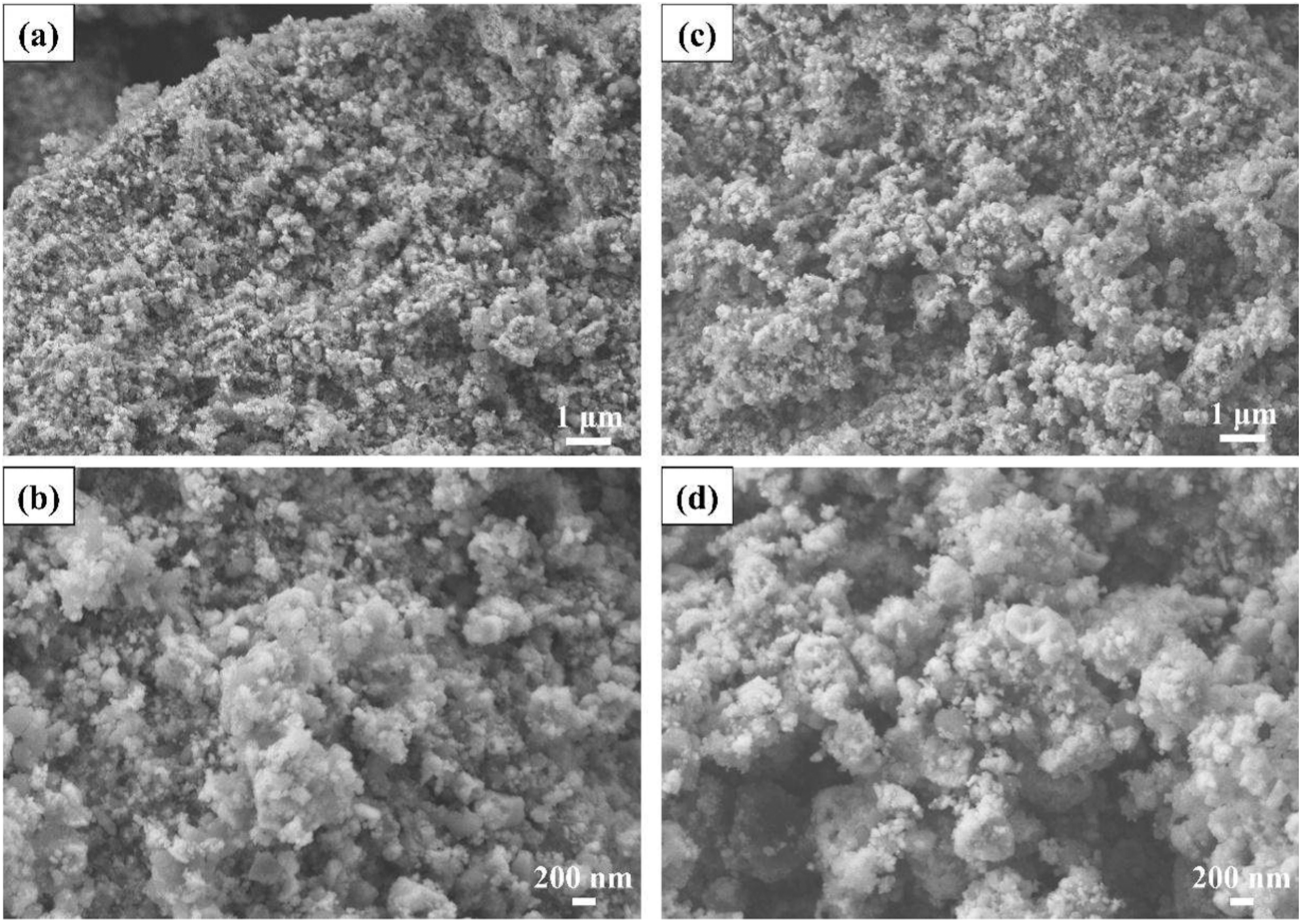
Secondary electron FE-SEM images of the synthesized composite powders: (a, b) ZB–10ZC, and (c, d) ZB–15ZC.

Figure 5 shows the secondary electron FE-SEM images of the synthesized composite powders. Low-magnification SEM images (Figure 5a,c) show that the ZrB_2_ and ZrC phases cannot be differentiated in terms of morphology, indicating a uniform mixture throughout the microstructure. High-magnification SEM images (Figure 5b,d) reveal that the particle sizes of the obtained composite powders are below 400 nm and that both samples are comparable. DLS analysis was conducted to minimize the effects agglomeration on particle size determination, as shown in Figure S3 of the SI file. The average particle sizes of the ZB–10ZC and ZB–15ZC samples were measured to be 249 and 330 nm, respectively. Furthermore, the average particle size of pure ZrB_2_ powders was measured as 300 nm, whose FE-SEM image and DLS analysis are presented in Figure S4 of the Supporting Information file. The size distributions shown in the histograms from the DLS analyses confirm that each powder exhibits a uniform particle size distribution within its respective sample. To compare the effects of different ZrC amounts in ZrB_2_–ZrC composites on the supercapacitor performance of the electrodes, it was important to achieve similar particle sizes to minimize the impact of particle size on the results. On the other hand, it is well-known that high surface areas can be achieved through composite design, and surface areas were measured using the BET technique. The BET surface area values for the ZB, ZB-10ZC, and ZB-15ZC samples are 7.44, 9.22, and 9.41 m^2^/g, respectively (Table S1, in the Supporting Information file). As expected, incorporating 15 wt % ZrC particles into the composites significantly enhanced the surface area.

### 3.2 Electrochemical Properties

The characterization results of the prepared samples, showing similar properties among the powders, allowed for meaningful performance tests. This facilitated a comparison between pure and composite powders with varying ZrC content and similar particle sizes, effectively evaluating their potential as electrode materials for supercapacitors. The PEIS, CV, and GCPL curves of symmetric supercapacitor devices fabricated with ZrB_2_ (ZB), ZrB_2_–10% ZrC (ZB-10ZC), and ZrB_2_–15% ZrC (ZB-15ZC) composite electrodes were analyzed at room temperature using a 6 M KOH aqueous electrolyte. The relationship between energy and power density was analyzed to determine the impact of varying ZrC content on the overall energy storage capabilities of the supercapacitor devices.

**Figure 6:**
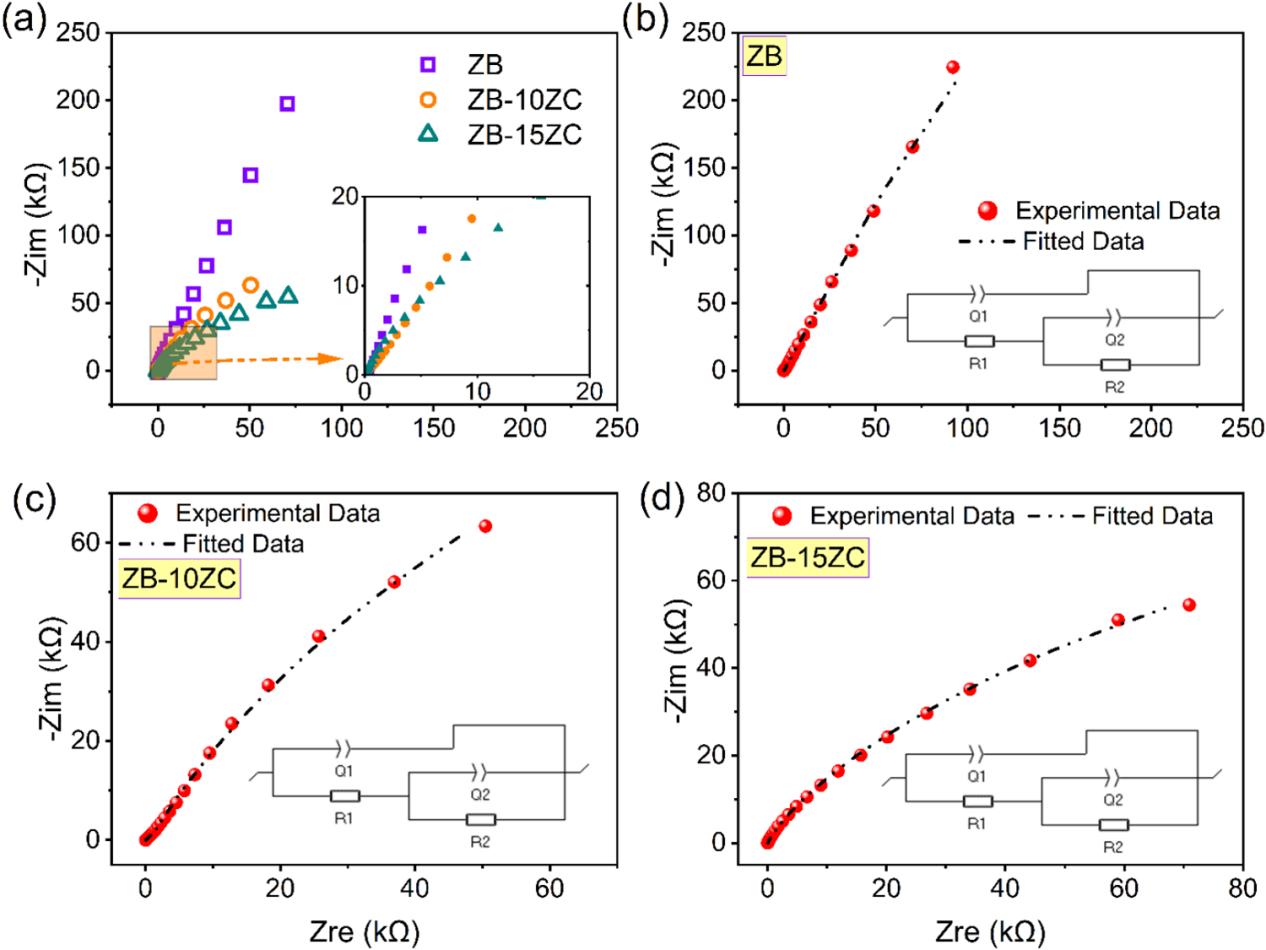
(a) Nyquist plots of all supercapacitor designs and *Z*_fit_ analysis results for the symmetric supercapacitors and equivalent circuits as inlet graphs (b) ZB, (c) ZB-10ZC, (d) ZB-15ZC.

The potentiostatic electrochemical impedance spectroscopy (PEIS) measurements were conducted to investigate the electrochemical properties of the synthesized materials (ZB, ZB-10ZC, and ZB-15ZC) in the frequency range from 1 MHz to 10 mHz. The obtained experimental data from the Nyquist plots were fitted using the *Z*_fit_ tool in the EC-Lab software. Figure 6b shows the fitted equivalent circuit elements, represented in the inlet graph of Figure 6b. The equivalent circuit elements are used to describe the various components that contribute to the overall impedance of the system, including the solution or series resistance. Figure 6a displays the Nyquist diagram for all the fabricated supercapacitors. The *x*-intercept of the Nyquist plot in the high-frequency range at the real part (*Z*_re_) is known to provide the solution or series resistance value.^36^ This resistance is associated with the electrolyte, current collector/electrode interface, and intrinsic resistance of the electrode material itself. When examining Figure 6a, it can be observed that the −*Z*_im_ value of ZB is higher compared to the ZrC-incorporated samples. Furthermore, in the presence of ZrC, there is an increase in the *Z*_re_ value compared to ZB. Thus, it is evident that ZB exhibits a higher double-layer capacitance behavior compared to the composite samples with different ZrC content. The increase in *Z*_re_ value is attributed to the resistances arising from the reactions occurring in the electrochemical cell in the presence of ZrC, which is an expected outcome. Adding ZrC to the structure has also increased the interfacial resistance between the electrodes.

The experimental data obtained from the Nyquist plots were fitted using the Z_fit_ tool in the EC-Lab software. The equivalent circuit used for fitting, shown in the inset of Figure 6b, consists of a resistor (*R*_1_) in series with a parallel combination of a constant phase element (CPE_1_, *Q*_1_) and a resistor (*R*_2_), which is further in series with another parallel combination of a constant phase element (CPE_2_, *Q*_2_) and a resistor (*R*_3_). *R*_1_ represents the equivalent series resistance (ESR), which includes contributions from the electrolyte resistance, contact resistance, and intrinsic resistance of the electrode material.^37,38^ The CPE elements (CPE_1_ and CPE_2_) are employed to model the nonideal capacitive behavior often observed in real electrochemical systems, which can arise from surface roughness, porosity, and inhomogeneous current distribution.^37,38^*R*_2_ and *R*_3_ represent resistances associated with interfacial processes, likely related to charge transport limitations at the electrode/electrolyte interface.

Table 3 presents the fitted values for the equivalent circuit elements. The increase in *R*_1_ with increasing ZrC content suggests an increase in the overall ESR, likely due to the lower conductivity of ZrC compared to ZrB_2_. The decrease in the magnitudes of CPE_1_ and CPE_2_ with ZrC addition indicates a decrease in the overall capacitance, which is consistent with the CV and GCPL results. The changes in *R*_2_ and *R*_3_ with ZrC content suggest that the interfacial processes are also affected by the composite composition.^39,40^

**Table 3:** Equivalent Circuit Parameters of Prepared Supercapacitors^a^

equivalent circuit (*Q*_1_/(*R*_1_ + *Q*_2_/*R*_*2*_))						
	*R*_1_	*Q*_1_	*R*_2_	*Q*_2_	*a*_1_	*a*_2_
ZB	7.8	83.9 × 10^–6^	9.4 × 10^6^	24.6 × 10^–6^	0.47	0.75
ZB-10ZC	55.5	46.2 × 10^–6^	0.3 × 10^6^	18.2 × 10^–6^	0.67	0.85
ZB-15ZC	165.5	20.92 × 10^–6^	0.4 × 10^6^	11.4 × 10^–6^	0.41	0.80

a 1 The unit of *R*_1_ and *R*_2_ is ohm, *Q*_1_ and *Q*_2_ is F·s^(a–1)^.

**Figure 7:**
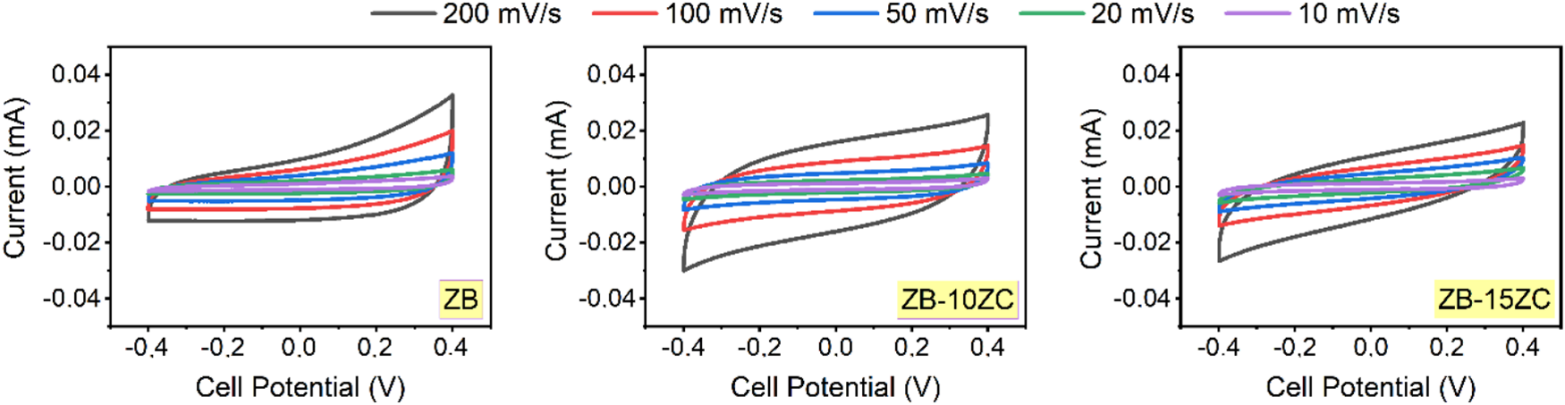
CV curves of fabricated supercapacitors at different scan rates.

Figure 7 shows the CV curves obtained from the supercapacitors, wherein ZB and ZB with different ZrC content composite-based electrodes were assembled. Upon examination of the CV curves, it is observed that all samples at various scan rates exhibit a rectangular-like profile during the charge/discharge process. As is known, this rectangular behavior indicates the characteristics of EDLC, signifying that charge storage occurs through the formation of electrical double layers at the electrode/electrolyte interface.^41^ When analyzing the current values of (ZB) and ZrC-added composite samples, it is seen that the addition of ZrC does not significantly affect the current values. As CV curves’ area under the curve corresponds to the specific capacitance of the supercapacitors and, consequently, the energy and power density, it is noteworthy that a partial decrease in the area under the CV curves is observed with the addition of ZrC.^42^ This decrease is assumed to be linked to the increase in ohmic and equivalent series resistance at the electrode/electrolyte interface due to ZrC addition, as observed in PEIS analysis.

While minor Faradaic reactions may occur, the dominant energy storage mechanism is EDLC, as evidenced by the rectangular CV curves (Figure 7). The observed increase in *Z*_re_ with ZrC addition (Figure 6a and Table 3) is likely due to several factors: increased interfacial resistance due to the introduction of ZrC particles; the lower conductivity of ZrC compared to ZrB_2_; possible surface passivation of ZrC in the alkaline electrolyte; and increased contact resistance between the active material and the current collector. These factors are more likely to contribute to the observed resistance increase than significant Faradaic reactions. The increase in interfacial resistance observed in the Nyquist plots and equivalent circuit fitting results suggests the potential formation of surface passivation layers due to ZrC addition. This behavior aligns with previous reports in the literature, where the incorporation of carbides into composite structures has been shown to influence the electrode/electrolyte interface by introducing resistive layers or modifying surface chemistry.^38^

The CV curves presented in Figure 7 exhibit a rectangular-like shape across various scan rates, which is characteristic of EDLC behavior. This indicates that the charge storage mechanism is primarily non-Faradaic, involving the adsorption and desorption of ions at the electrode/electrolyte interface. The absence of distinct redox peaks in the CV curves further supports the dominance of EDLC over pseudocapacitive processes, suggesting minimal contribution from Faradaic reactions. This behavior is typical for materials like ZrB_2_ and ZrC, which are known for their high conductivity and stability, making them suitable for EDLC applications. The addition of ZrC to the ZrB_2_ influences the specific capacitance by altering the surface area of the composite electrodes. As already reported in Table S1 (in the Supporting Information file), the surface area increased from 7.44 to 9.41 m^2^/g after incorporating 15 wt % ZrC into ZrB_2_. ZrC’s incorporation can lead to changes in the microstructure, potentially increasing the surface roughness of the electrode material. This increased surface area can enhance ion accessibility and facilitate faster ion transport to the electrode surface, which is crucial for charge storage. However, the presence of ZrC may also introduce additional interfacial resistance and passive layers, which can reduce the effective surface area available for double-layer formation, thereby impacting the overall capacitance. These microstructural changes highlight the complex interplay between increased surface roughness and the potential for increased resistive losses, which must be carefully balanced to optimize the electrochemical performance of the supercapacitors.

**Figure 8:**
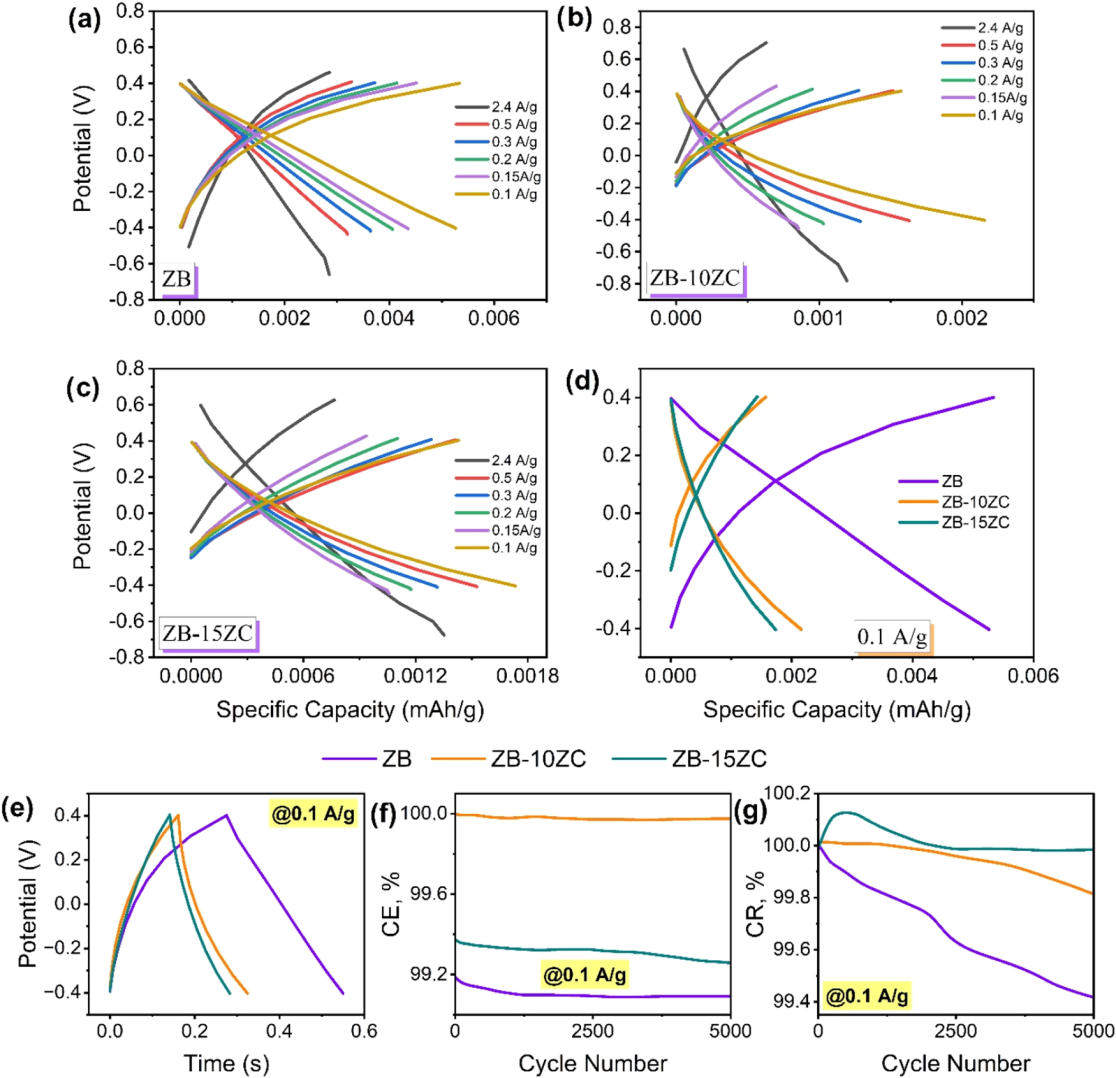
GCPL profiles of the symmetric supercapacitors at (a–c) different current densities, (d) current density of 0.1 A/g, (e) time-dependent potential profiles of fabricated supercapacitor at the first cycle, (f) comparison of Coulombic efficiency (CE, %) and (g) capacitance retention (CR, %).

The galvanostatic charge–discharge (GCPL) profiles shown in Figure 8 further corroborate the EDLC mechanism. The linear voltage–time relationship observed during the charge–discharge cycles is indicative of a capacitive behavior, where the charge storage is governed by the formation of an electric double layer rather than Faradaic redox reactions. The slight IR drop observed in the GCPL curves is consistent with the equivalent series resistance (ESR) values obtained from the impedance analysis, which reflects the intrinsic resistance of the electrode materials and the electrolyte.

The addition of ZrC to the ZrB_2_ appears to influence the electrochemical performance by modifying the surface area and conductivity of the composite electrodes. While the specific capacitance and energy density decrease with increasing ZrC content, the power density shows a notable increase. This suggests that the addition of ZrC enhances charge–discharge kinetics, likely by increasing the surface area, thereby improving ion transport and reducing diffusion pathways within the electrode structure. The increased power density is particularly advantageous for applications requiring rapid energy delivery. Consequently, the electrochemical behavior observed in the CV and GCPL analyses highlights the potential of ZrB_2_–ZrC composites as high-performance electrode materials for supercapacitors. The balance between energy and power density can be further optimized by tailoring the composite composition and microstructure, providing a pathway for the development of advanced energy storage devices.

Figure 8 shows fabricated supercapacitor devices’ galvanostatic charge–discharge profiles. The graphs of specific capacity vs potential for each supercapacitor design at different and 0.1 A/g current densities are shown in Figure 8a–d. The specific capacity represents the supercapacitor’s charge amount per unit mass.^43^ Considering different current densities, the highest specific capacity for ZB and ZrC-incorporated supercapacitor designs was obtained at 0.1 A/g current density. Additionally, when examining their behaviors at various current densities, they exhibited a similar trend, with specific capacity decreasing at 0.5, 0.3, 0.2, and 0.15 A/g (i.e., with decreasing current densities). At the current density of 0.1 A/g, where the highest specific capacity was achieved, the specific capacity for ZB, ZB-10ZC, and ZB-15ZC designs were determined as 0.0052, 0.0021, and 0.0017 mAh/g, respectively. The increase in ZrC addition was observed to lead to a partial decrease in the specific capacity, indicating a reduction in the charge storage capacity of the supercapacitor. This decrease can be attributed to the resistive losses and charge losses resulting from the reactions occurring in the presence of ZrC. Adding ZrC introduces some resistance and charge loss mechanisms, possibly contributing to the observed decrease in specific capacity. The absence of distinct voltage plateaus indicates a non-Faradaic capacitive behavior dominant in the charge storage mechanism.^44^ This behavior is characteristic of EDLCs, where charge storage occurs mainly through the adsorption of ions at the electrode–electrolyte interface. Moreover, the excellent cycling stability observed in all fabricated supercapacitors designed over 5000 cycles at a current density of 0.1 A/g demonstrates their robust electrochemical performance.^45^

Figure 8e–g presents the time-dependent potential profiles, Coulombic efficiency, and capacitance retention values for the prepared supercapacitor designs obtained from the GCPL analysis. As shown in Figure 8e, the charge/discharge period for the ZB sample was approximately 0.27 s, while this time decreased to 0.16 and 0.14 s with increasing ZrC addition. The reduction in charge–discharge times gives information about an enhancement in the performance of the supercapacitor and could be particularly significant for high-power applications. Rapid charge–discharge times imply a higher response capability of the supercapacitor, thereby enhancing its ability to meet instant energy demands.^46^ Additionally, a slight decrease in internal resistance (IR drop) was observed in the charge/discharge curves. This drop is attributed to increased equivalent series resistance (ESR) due to various factors. These resistances contribute to the overall internal resistance of the supercapacitor, affecting its performance and efficiency during charge and discharge cycles.^47,48^ The PEIS analysis consistently revealed an increase in equivalent series resistance with ZrC addition, confirming the findings obtained from the GCPL analysis.

Figure 8f,g presents the percentage of Coulombic efficiency (CE, %) and capacitance retention (CR, %) values for the prepared supercapacitor designs after 5000 cycles. After 5000 cycles, all designed supercapacitors exhibited approximately 100% CR. The values for ZB, ZB-10ZC, and ZB-15ZC samples were calculated as 99.4, 99.8, and 99.9%, respectively, indicating excellent cyclic stability. The increasing CR (%) signifies improved performance and reduced capacity loss of the supercapacitor during long-term usage. CE (%) represents the ratio of utilized and produced electrical charge during an electrochemical reaction.^49^ As shown in Figure 8f, with 10% ZrC, the CE slightly increased, while at 15% ZrC (ZB-15ZC sample), it decreased to 99.1%. The decrease in CE observed with the addition of ZrC may not be primarily due to increased interface resistance, as the data in Table 3 suggest a decrease in interface resistance. Instead, this decrease could be attributed to changes in the electrode surface chemistry or the formation of a passivation layer, which can affect charge transfer processes. The specific chemical reactions responsible for these changes are not explicitly identified in this study, but they could involve side reactions between the electrode material and the electrolyte, leading to the formation of surface films or other byproducts that impact the charge–discharge efficiency. The results demonstrate that the designed supercapacitors exhibited exceptional capacitance retention and cyclic stability even after 5000 cycles. The CE, influenced by the ZrC addition, suggests the occurrence of certain electrochemical reactions and increased equivalent series resistance at the electrode interfaces. Besides, the cycling tests were conducted for 5000 cycles, as both the Coulombic efficiency (CE) and capacitance retention (CR) values exhibited minimal degradation, with CR values of 99.4, 99.8, and 99.9% for ZB, ZB-10ZC, and ZB-15ZC samples, respectively. These results indicate excellent cyclic stability and durability of the fabricated supercapacitors. Given the high stability observed after 5000 cycles, further cycling tests were not performed in this study. However, we acknowledge the importance of extended cycling tests for practical applications and will address this in future studies to further validate the long-term performance of the electrodes.

The primary energy storage mechanism of the ZrB_2_–ZrC composite is electric double-layer capacitance (EDLC). This conclusion is supported by the rectangular-like shape of the CV curves at various scan rates (Figure 7), indicative of a non-Faradaic charge storage process. The absence of distinct redox peaks in the CV curves further confirms the limited contribution of Faradaic reactions. Additionally, the linear voltage–time relationship observed in the GCPL profiles (Figure 8) is characteristic of EDLC behavior. The addition of ZrC appears to influence electrochemical performance as evidenced by the changes in specific capacitance, energy density, and power density with varying ZrC content (Table 4). However, the fundamental EDLC mechanism remains unchanged. The increased equivalent series resistance observed with ZrC addition in the PEIS analysis (Figure 6 and Table 3) correlates with the decrease in specific capacity observed in the GCPL data, further supporting the influence of ZrC on the charge storage efficiency.

**Table 4:** Electrochemical Parameters of Fabricated Supercapacitors

design	specific capacitance (mF/g)	energy density (Wh/kg)	power density (W/kg)
ZB	79.5	8.8	118
ZB-10ZC	59.5	6.6	149
ZB-15ZC	52.3	5.8	155

The electrochemical properties of the fabricated supercapacitors are detailed in Table 4, aligning well with the CV and PEIS outcomes. The ZB sample exhibits a specific capacitance of approximately 79 mF/g, with energy and power densities of 8.8 Wh/kg and 118 W/kg, respectively. Introducing ZrC results in a decrease in specific capacitance, with the ZB-15ZC sample dropping to about 52.3 mF/g, and a corresponding reduction in energy density. This decline can be attributed to changes in the electrode surface chemistry and the formation of passive layers, which reduce active sites for charge storage and increase equivalent series resistance. These modifications potentially alter the electrochemical interface, leading to increased resistive losses.

Conversely, the addition of ZrC significantly enhances power density, as evidenced by the GCPL analysis. The reduction in charge/discharge times improves the supercapacitor’s response capability, contributing to the observed increase in power density. Impedance analysis further supports these findings, showing reductions in both resistance (*Z*_re_) and reactive components (−*Z*_im_) with ZrC incorporation, indicating more rapid ion displacement on the electrode surface.

**Figure 9:**
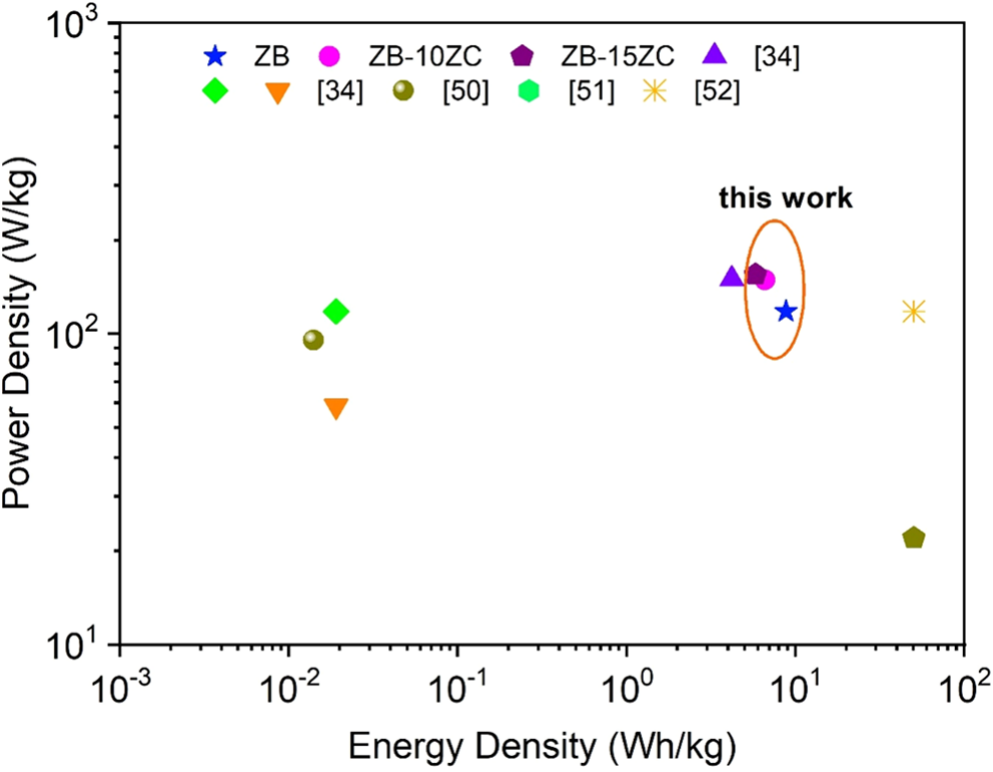
Ragone plot comparing devices with others in the literature.

A Ragone plot, illustrating the relationship between energy density and power density for the fabricated supercapacitors, is presented in Figure 9.^34,50−52^ The ZrB_2_–ZrC composite electrodes exhibit significantly higher power density compared to pure ZrB_2_, with the ZB-15ZC sample achieving a peak power density of 155 W/kg. However, this improvement in power density is accompanied by a slight reduction in energy density, as expected due to the trade-off between these parameters. The ZB-15ZC sample demonstrates an energy density of 5.8 Wh/kg, which is still within a competitive range for high-power supercapacitor applications. These results highlight the potential of ZrB_2_–ZrC composites as promising electrode materials for applications requiring rapid energy delivery and long-term stability.

## 4 Conclusions

In conclusion, this research highlights the potential of boride and carbide-based materials for energy storage applications, focusing on the synthesis of pure zirconium boride (ZrB_2_) and zirconium carbide (ZrC) compounds, as well as their composites. We achieved high-purity composite powders with submicron particle sizes and high surface areas using a mechanical activation-assisted method followed by heating. Characterization techniques, including XRD, SEM, DLS, and BET confirmed the quality and scale of the synthesized materials. The supercapacitor devices incorporating these powders as symmetrical electrodes demonstrated impressive energy densities between 5.8 and 8.8 Wh/kg. Notably, the ZrB_2_–15 wt % ZrC composite reached a peak power density of 155 W/kg, outperforming the pure ZrB_2_ sample’s 118 W/kg. Although the energy density decreases with increasing ZrC content, the power density significantly improves. This enhancement is likely due to the increased surface area of composite structures, which facilitates ion transport and shortens diffusion pathways within the electrode structure, thereby boosting power density. Moreover, the cycling tests showed remarkable capacitance retention and stability, with 99.9% retention after 5000 cycles, signifying the durability of prepared composites. Overall, the ZrB_2_–ZrC composites exhibit superior energy and power densities and excellent cycling performance, establishing them as promising candidates for high-performance supercapacitor applications.

## Supporting Information Available

DTA/TG and SEM/EDX analyses of the synthesized ZB–10ZC composite powders; DLS particle size measurements of the synthesized composite powders; microstructure and particle size measurements of the synthesized pure ZrB_2_ powders; and BET surface area values of the powders (PDF)
