# Supplementary material for: Durable ZrB2–ZrC Composite Materials as Advanced Electrodes for High-Performance Supercapacitors
Source: ACS Omega. 2025 Apr 25;10(17):18073–84. doi: 10.1021/acsomega.5c01560 (PMC12060047; doi:10.1021/acsomega.5c01560)
Supplement: Supplementary file 1 — ao5c01560_si_001.pdf [file ao5c01560_si_001.pdf]

Durable ZrB_2 – ZrC composite materials as advanced electrodes for high – performance supercapacitors

Aybike Paksoy[1+], Ahmet Güngör[2+], İpek Deniz Yıldırım[2], Seyedehnegar Arabi[3], Emre Erdem[2*], Özge Balcı-Çağıran[4*]

[1]Koç University Boron and Advanced Materials Application and Research Center (KUBAM), Rumelifeneri Yolu, Sarıyer, 34450 İstanbul, Turkey

[2]Faculty of Engineering and Natural Sciences, Sabancı University, Orhanlı, Tuzla 34956, İstanbul, Turkey

[3]Industrial Systems Engineering Department, University of Regina, Regina, Canada

[4]İzmir Institute of Technology, Department of Materials Science and Engineering, 35430 Urla, İzmir, Turkey

*Corresponding Authors: emre.erdem@sabanciuniv.edu and ozgebalci@iyte.edu.tr

[+]These authors contributed equally to this work.

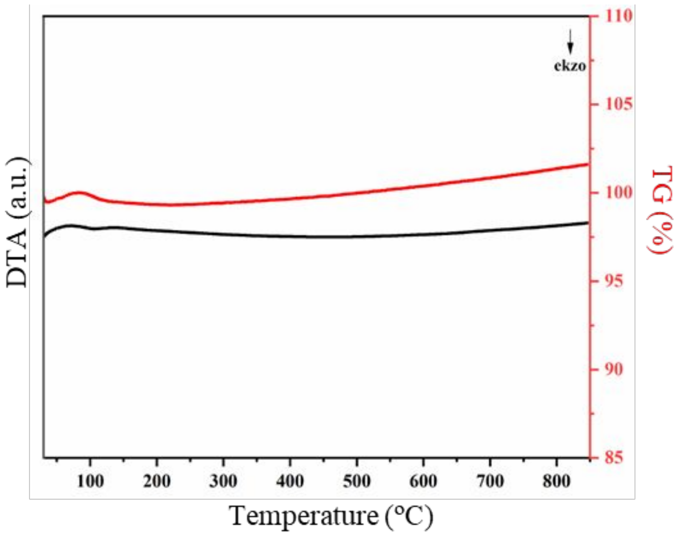

Figure S1. DTA/TG analysis of the synthesized ZB–10ZC composite powders.

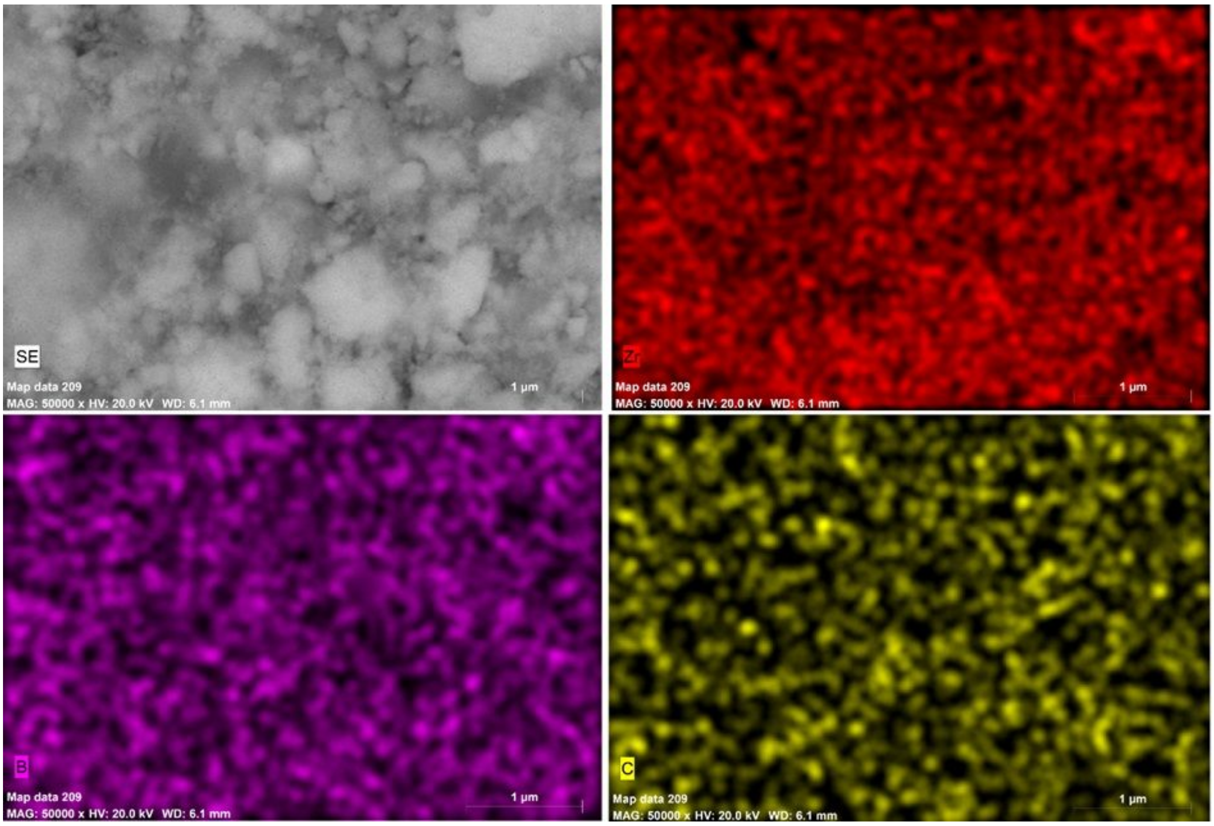

Figure S2. SEM/EDX analysis of the synthesized ZB–10ZC composite powders.

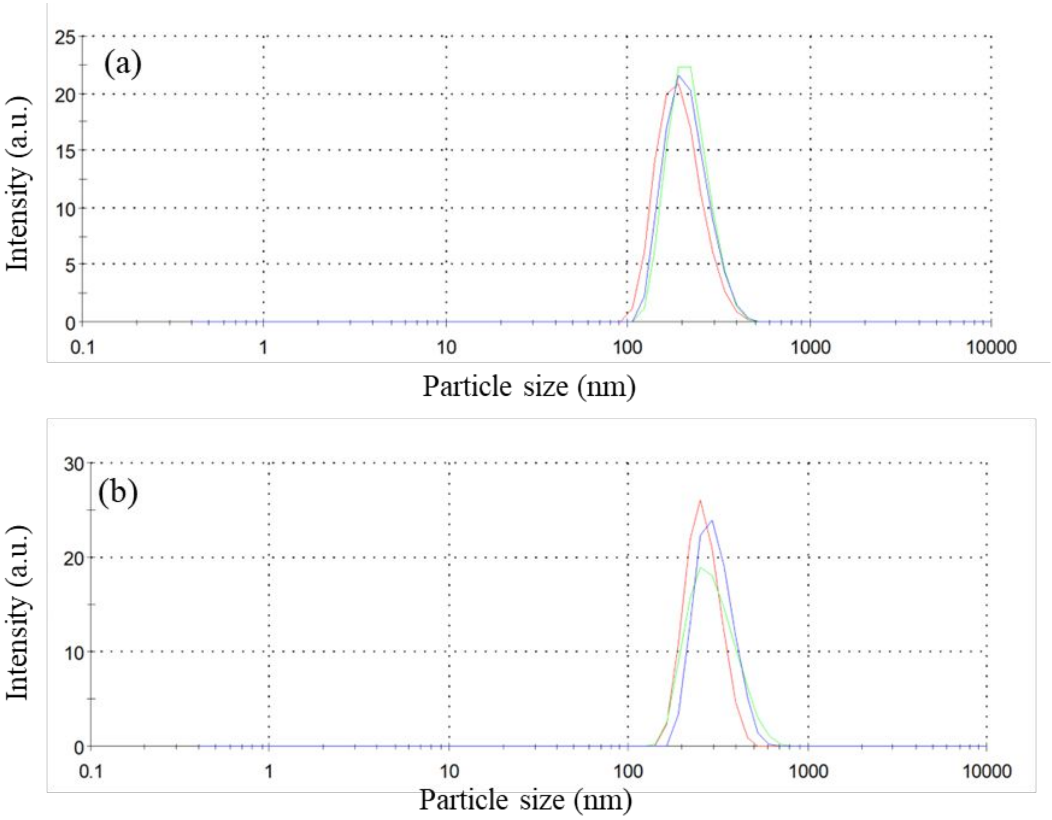

Figure S3. DLS particle size measurements of the synthesized composite powders: (a) ZB–10ZC, and (b) ZB–15ZC. The average particle sizes of the ZB–10ZC and ZB–15ZC samples were measured to be 249 nm and 330 nm, respectively.

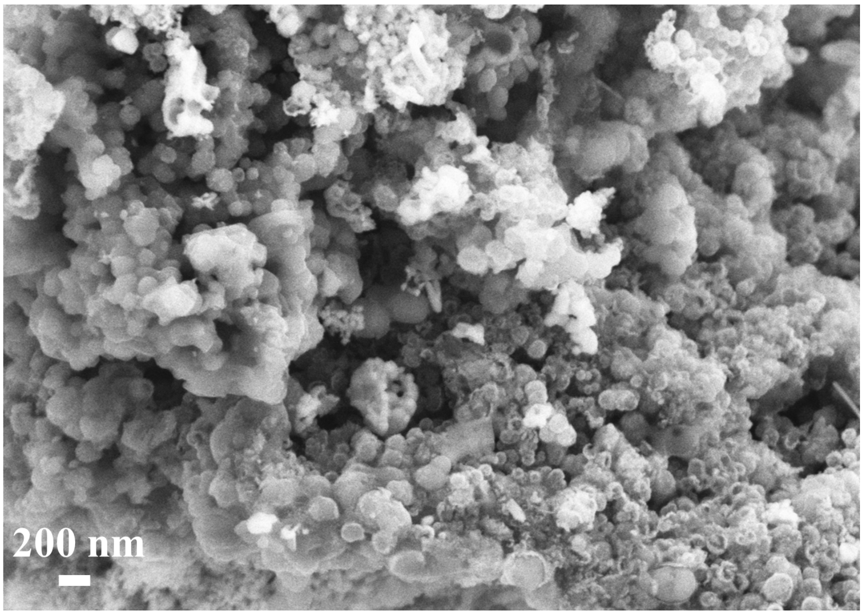

(a)

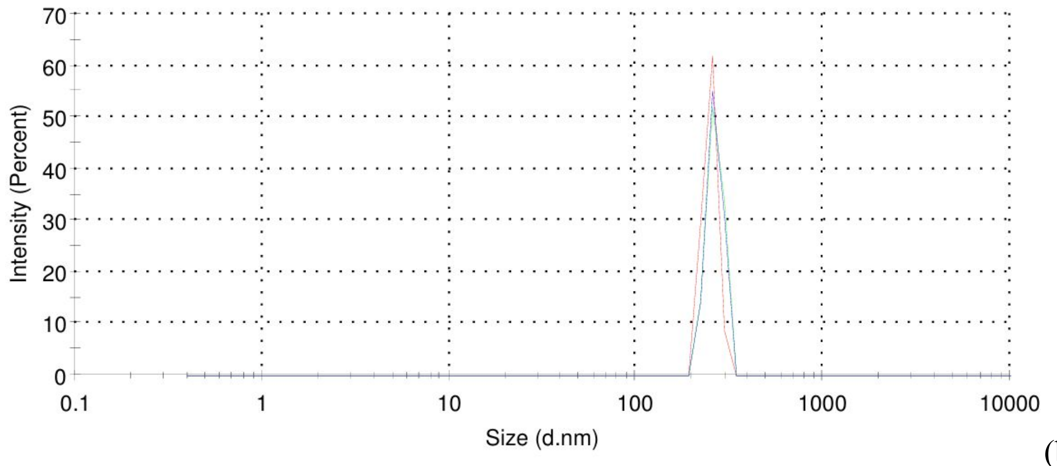

(b)

Figure S4. Microstructure and particle size measurements of the synthesized pure ZrB_2 powders: (a) FE-SEM image and, (b) DLS particle size measurement. The average particle size of the ZrB_2 sample was measured to be 300 nm.

Table S1. BET surface area values of the powders.

Sample	BET surface area (m^2/g)
**ZB**	7.44
**ZB-10ZC**	9.22
**ZB-15ZC**	9.41
